# Plant-soil feedbacks from 30-year family-specific soil cultures: phylogeny, soil chemistry and plant life stage

**DOI:** 10.1002/ece3.1487

**Published:** 2015-05-22

**Authors:** Zia Mehrabi, Thomas Bell, Owen T Lewis

**Affiliations:** 1 Department of Zoology, University of OxfordSouth Parks Road, Oxford, OX1 3PS, UK; 2 Imperial College LondonSilwood Park Campus, Buckhurst Road, Ascot, Berkshire, SL5 7PY, UK

**Keywords:** Aboveground–belowground ecology, coexistence, germination, Janzen-Connell, Oxford University Botanic Gardens, pathogens, phylogeny, seedling, soil sickness

## Abstract

Intraspecific negative feedback effects, where performance is reduced on soils conditioned by conspecifics, are widely documented in plant communities. However, interspecific feedbacks are less well studied, and their direction, strength, causes, and consequences are poorly understood. If more closely related species share pathogens, or have similar soil resource requirements, plants may perform better on soils conditioned by more distant phylogenetic relatives. There have been few empirical tests of this prediction across plant life stages, and none of which attempt to account for soil chemistry. Here, we test the utility of phylogeny for predicting soil feedback effects on plant survival and performance (germination, seedling survival, growth rate, biomass). We implement a full factorial experiment growing species representing five families on five plant family-specific soil sources. Our experiments exploit soils that have been cultured for over 30 years in plant family-specific beds at Oxford University Botanic Gardens. Plant responses to soil source were idiosyncratic, and species did not perform better on soils cultured by phylogenetically more distant relatives. The magnitude and sign of feedback effects could, however, be explained by differences in the chemical properties of “home” and “away” soils. Furthermore, the direction of soil chemistry-related plant-soil feedbacks was dependent on plant life stage, with the effects of soil chemistry on germination success and accumulation of biomass inversely related. Our results (1) suggest that the phylogenetic distance between plant families cannot predict plant–soil feedbacks across multiple life stages, and (2) highlight the need to consider changes in soil chemistry as an important driver of population responses. The contrasting responses at plant life stages suggest that studies focusing on brief phases in plant demography (e.g., germination success) may not give a full picture of plant–soil feedback effects.

## Introduction

The mechanisms structuring plant diversity within ecological communities have been the focus of extensive study and debate (e.g., de Candolle 1820; Janzen 1970; Gentry 1988; Hubbell 2000). Most plants are highly intimate with soil, and it is increasingly realized that plant–soil interactions may be key for explaining variations in plant community diversity and species composition (e.g., Fitter 2005; Bardgett and Wardle 2010; Fukami and Nakajima 2013; van der Putten et al. 2013).

It is often suggested that closely related species compete more strongly and are therefore less likely to coexist than distant relatives (Darwin 1859; MacArthur and Levins 1967). Negative fitness consequences of growing close to related species can arise in at least two ways through conditioning of the soil (a phenomenon known as negative plant–soil feedbacks). First, soil-borne pathogens can drive plant–soil feedback effects (e.g., Augspurger 1984; Reinhart and Clay 2009; Bagchi et al. 2010). As more closely related plant species often share natural enemies, plants growing in close proximity to related species may suffer lower performance or survivorship (Freckleton and Lewis 2006; Silvertown et al. 2006; Webb et al. 2006; Gilbert and Webb 2007). Second, plant–soil feedbacks could be generated through soil chemistry (Finzi et al. 1998; Chapman et al. 2006; Bonanomi et al. 2008; Bever et al. 2010; McCarthy-Neumann and Kobe 2010a,b): if more closely related species share resource requirements, competition for soil nutrients will make it advantageous to grow on soil used by more distant relatives. However, if the dissimilarity in traits that influence soil chemistry changes is not related to the phylogenetic distance separating species, then phylogenetic distance may be a poor predictor of plant–soil feedback effects, even if soil chemistry changes influence plant—soil feedbacks. Although the effect of phylogenetic distance on plant–soil feedbacks has been reported (Brandt et al. 2009; Burns and Strauss 2011; Liu et al. 2012), recent work questions whether the phylogenetic distance separating species can be used to predict the outcome of species interactions through the soil in any general way (Mehrabi and Tuck 2015). Nevertheless, there has been a lack of plant–soil feedback studies testing this prediction which track plant survival and performance responses (e.g., seedling germination, mortality, growth, and biomass together) and none that attempt to account for soil chemistry effects (Ehrenfeld et al. 2005; Bezemer et al. 2006; Harrison and Bardgett 2010).

In this article, we test the hypothesis that the strength and direction of plant–soil feedbacks are related to phylogenetic distance separating species in different plant families. We do this by reciprocally growing representative species from five plant families on soil sources cultured for over 30 years in plant family-specific beds at Oxford University Botanic Gardens. Our experiments generate data on plant survival and performance under 30 pairwise plant–soil combinations and include multiple life stages to test for effects at different stages of root development and plant growth. In addition, we test whether soil chemistry distances between soils are better able to explain feedback response than phylogenetic distances.

## Materials and Methods

### Study system

**Figure 1:**
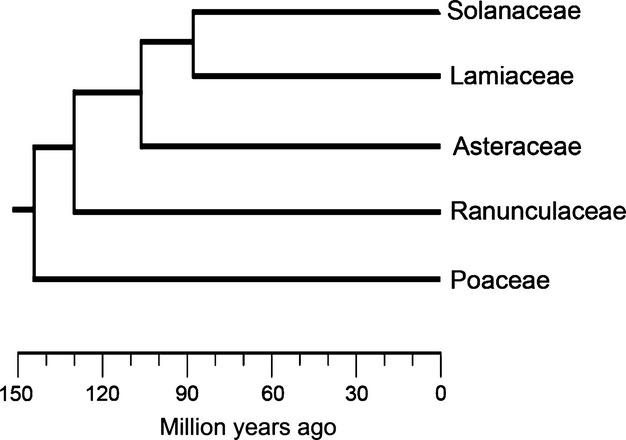
Sister relationships between plant families used in the study. Relationships and node ages were extracted from the strict consensus angiosperm supertree of Davies et al. (2004) using the AGENODE function in Phylocom 4.1 (Webb and Donoghue 2005).

In January 2010, we collected soil from plant beds at Oxford University Botanic Garden (51°45′4.45″ N, 1°14′48.78″ W) dedicated to five plant families (Solanaceae, Lamiaceae, Asteraceae, Ranunculaceae, and Poaceae) that have hierarchical sister relationships (Fig.1; Davies et al. 2004). Approximately 20 L of soil was collected to a depth of 10 cm from a single bed for each plant family. Beds for soil sampling were determined from within family bed groups in the garden based on availability at the time of sampling (e.g. those that were bare during winter). The same families are known to have been cultivated on the same beds for at least 30 years (Timothy Walker, Director Oxford University Botanic Gardens, pers. comm.). No pesticides have been applied since the 1980s and all beds have been treated identically, with only occasional and equal application of compost or small quantities of NPK fertilizer. The horticultural setup and close proximity of the beds mean that unmeasured environmental conditions were unlikely to confound the effect of family culture, although species richness of the family cultures was not constant across family beds (Fig. S1). The lack of replication at the bed level in our design limits the inference from our experiments to the beds used in this study. Soil was stored at ambient temperatures (to match those in the plant beds) until the start of the experiments that took place at the John Krebs Field Station, Wytham, Oxfordshire, UK (51°46′58.70″ N, 1°18′57.37″ W), during April–July 2010.

### Feedback effects

To investigate the strength and specificity of plant–soil feedback effects, species representing each family (Table1) were grown reciprocally on soil from each family-specific plant bed. The species chosen were all annuals, to minimize variation in life-history effects (see Tofts and Silvertown 2002; Brandt et al. 2009) and to maximize plant–soil feedback responses (Kulmatiski et al. 2008). With the exception of *M. recutita* (used for availability of seed at time of experiment), all the focal species have been grown at the Oxford University Botanic Garden over the last 15 years, along with other confamilial species. Some variation in species placement across beds has occurred, and not all species have been grown every year (Oxford University Botanic Garden, unpubl. data). A full factorial design was employed to test the effects of each soil on the growth of each species, involving 30 pairwise comparisons over 300 plant pots (6 plant species × 5 soil family beds × 10 replicates). *Petunia integrifolia* was included in the experiment to provide an additional representative for the Solanaceae, because seed of *N. tobacum* (the initial focal species) did not germinate within the first 21 days. Soil from each family source was separately homogenized by hand mixing and potted in 12-cm-diameter plastic plant pots after excluding coarse roots and stones. Seven seeds were sown in each pot beside uniquely numbered wooden toothpicks, and their performance tracked. Pots were arranged in random positions within blocked rows across a bench in an unheated greenhouse. Pot positions were rotated within rows at weekly intervals. Pots were watered from above up to twice daily, as required, and nonfocal species were weeded weekly. The germination success of 2100 individual seeds and seedling mortality was recorded over 21 days at weekly intervals. At 21 days, plants were thinned to a maximum of three individuals per pot to reduce intraspecific competition. Plant height was measured on days 21, 35, 49, and 81. After 12 weeks (prior to senescence), the experiment was terminated by destructive harvest of aboveground biomass. Plant material from each pot was amalgamated, and samples were dried to constant dry mass at 70°C and then weighed to 0.00001 g on a microbalance (Kern ABT 220-5DM, Balingen, Germany).

**Table 1:** Species used in the study. All seeds were of commercial quality (B&T World Seeds, Paguignan, France). Seed masses are taken from supplier's best estimates

Species	Family	Life history	Seed mass (g)
Dicots			
* Petunia integrifolia* Juss.	Solanaceae	Annual	5 × 10^−5^
* Nicotiana tobacum* L.	Solanaceae	Annual	5 × 10^−5^
* Salvia viridis* L.	Lamiaceae	Annual	1.7 × 10^−3^
* Matricaria recutita* L.	Asteraceae	Annual	6 × 10^−5^
* Nigella damascana* L.	Ranunculaceae	Annual	2.5 × 10^−4^
Monocots			
* Triticum aestivum* L.	Poaceae	Annual	3.6 × 10^−2^

### Soil chemical analysis

To measure base cation and nitrogen content, 10 subsamples of each family-specific soil source were amalgamated, dried in the sun, and passed through a 2-mm sieve. Thirty grams of samples was tested for exchangeable base cations (Ca, K, Mg, and Na) using BaCl_2_ extraction, and for total nitrogen and carbon content. Analyses were carried out by the Chemical Analysis Service of the Centre for Forestry and Climate Change, Surrey, UK.

### Data analysis

Feedback in germination success, seedling mortality, growth rate, and mean plant biomass was calculated as follows:

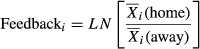
 where 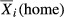 is the mean response of a focal species on soil from its own family and 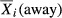 is the mean response of a focal species on soil cultured by a different family. For mortality, the denominator and numerator were inversed for consistency in feedback sign interpretation. The probability of a focal species germinating or dying was calculated for each replicate pot prior to calculating the ratio of the mean effects of home and away (e.g., equivalent to LN relative risk ratio). Height measures were used in growth rate analyses to reflect resource acquisition and allocation to photosynthesis (Brandt et al. 2009). Plots of mean height against time were exponential (except for *T. aestivum*), so the gradient of the slope relating log height to time was used as a proxy growth rate index. *Petunia integrifolia* was omitted from growth rate analyses because of late germination. Taking the natural logarithm of the ratio of mean plant responses on home versus away soils creates a linearized feedback index with desirable statistical properties that is intuitively bound between 1 and −1, where positive values correspond to positive plant–soil feedback (i.e., performing better on home vs. away soil) and negative values correspond to negative plant–soil feedback (i.e., performing better on away soil vs. home soil).

Pairwise phylogenetic distances for each pair of families were estimated using Phylomatic (Webb and Donoghue 2005). Hypothesized relationships among families were based on Davies et al. (2004). The PHYDIST function of Phylocom 4.1 (Webb and Donoghue 2005) was then used to extract pairwise phylogenetic distance between species.

Principal component analysis was performed on soil chemistry parameters (Ca, K, Mg, Na, and C:N), to derive an axis representing linear combinations of weighted soil chemistry. The first principle component (explaining 70% of the variance in soil chemistry, eigenvalue: 3.49) was extracted and used in the analysis, with negative and positive values indicating an increase in soil C:N and an increase in exchangeable base cations, respectively.

Feedback indices for each plant response were analyzed using linear mixed models, with phylogenetic distance (or soil chemistry change) as a fixed effect and focal species as a random effect (to account for the dependencies from using common control values in calculation of feedback indices), using the *lme4* package in R 3.0.2. Separate models were fit for the response variables biomass, growth rate, seed germination, and seedling mortality. The importance of phylogeny (or soil chemistry) for predicting differences in feedback responses was tested using likelihood ratio tests, comparing the fit of these models to null models with the phylogenetic (or soil chemistry) distance term removed. A full dataset for this publication is deposited on Dryad Digital Repository (Mehrabi 2015).

## Results

**Figure 2:**
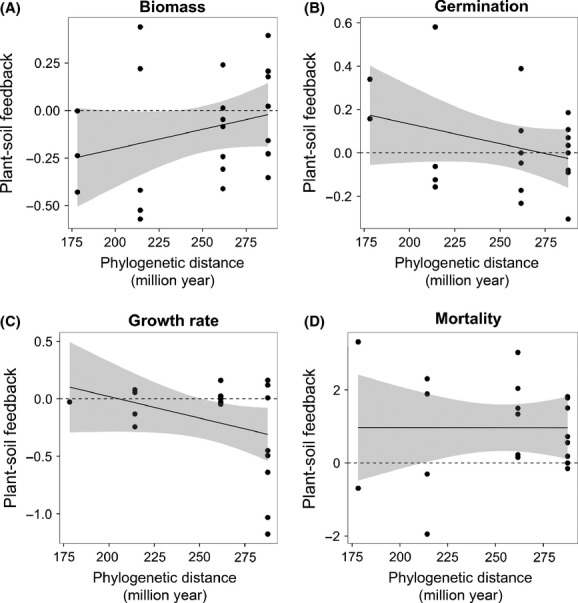
Effects of phylogenetic distance on the strength of plant–soil feedbacks. The relationship between phylogenetic distance between plant–soil interactions on strength of plant–soil feedback measured as (A) dry shoot biomass (g), (B) probability of germination, (C) growth rate (log mm/week) and (D) probability of seedling mortality. Feedback is calculated as follows: 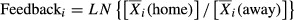, where 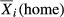 is the mean response of a focal species on soil from its own family and 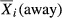 is the mean response of a focal species on soil cultured by a different family. For mortality, the denominator and numerator were inversed for consistency in interpretation (e.g., positive feedback is when a species responds better on its own families soil and negative feedback when it responds better on a soil from a different family). All means were calculated from 10 replicates. Unlike the estimates shown in the text, the plotted regression slope and 95% intervals assume a fixed model with no sampling dependence.

Phylogenetic distance between plant–soil interactions was a weak positive predictor of the magnitude of feedback effects for biomass responses (slope: 0.0029 ± 0.0011 SE; 1.0029 times more biomass accumulated on home soils vs. away soils for each million years distance separating home and away soils) (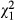 = 5.17, *P* = 0.02) (Fig.2A), and a marginal and weak negative predictor of germination responses (slope: −0.0017 ± 0.0009 SE; germination was 1.002 times as likely to occur on away vs. home soils for each million years in distance separating home and away soils) (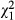 = 3.41, *P* = 0.06) (Fig.2B). However, phylogeny did not help explain variation in mortality (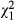 = 0.02, *P* = 0.88), or growth rate (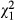 = 0.08, *P* = 0.77) (Fig.2C–D).

**Figure 3:**
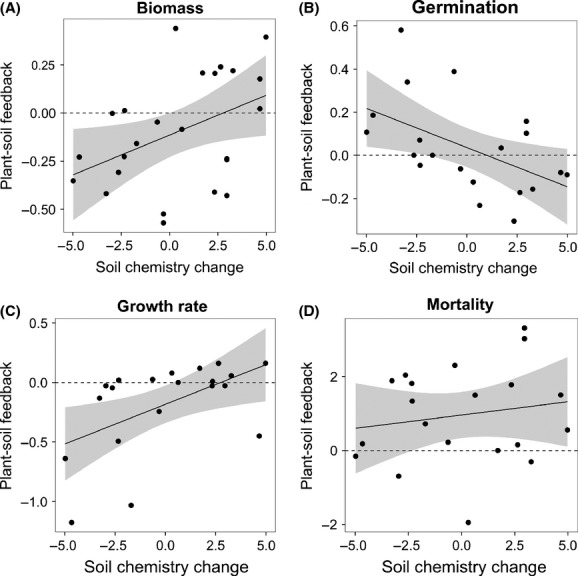
Effects of soil chemistry on plant–soil feedbacks across life stages. The relationship between changes in soil chemistry between plant–soil interactions on strength of plant–soil feedback measured as (A) dry shoot biomass (g), (B) probability of germination, (C) growth rate (log mm/week) and (D) probability of seedling mortality. Feedback was calculated as in Fig.2. Soil chemistry scores are derived from a linear combination of abiotic parameters, where positive scores represent increasing base cation content (Ca, K, Na, and Mg mg/kg) and negative scores increasing C:N ratio. All means were calculated from 10 replicates. Unlike the estimates shown in the text, the plotted regression slope and 95% intervals assume a fixed model with no sampling dependence.

Plants accumulated more biomass on soils with higher base cation content (Ca, K, Na, and Mg) and lower C:N ratios (slope: 0.067 ± 0.016 SE; 1.069 times more biomass accumulated per unit of soil chemistry change) (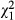 = 11.55, *P* < 0.001) (Fig.3A). Soil chemistry was also a better predictor of biomass feedback effects than phylogenetic distance (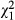 = 5.84, *P* < 0.0001). The effects of soil chemistry on germination success followed the reverse pattern, with higher germination rates on soils with higher C:N ratios and lower base cation content (slope: −0.038 ± 0.013 SE; 1.038 times as likely to occur for each unit of soil chemistry change) (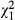 = 7.7, *P* < 0.01) (Fig.3B); with phylogeny, soil chemistry was also a better predictor of germination feedback effects (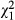 = 4.28, *P* < 0.0001). Soil chemistry was not a significant explanatory variable for seedling mortality (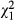 = 0.72, *P* = 0.39) or growth rate (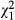 = 1.01, *P* = 0.31) (Fig.3C–D).

## Discussion

Our study investigated the effects of plant phylogeny on plant–soil feedbacks across multiple life stages. We investigated whether variance in feedback effects was related to the phylogenetic distance separating families and whether these plant soil feedback effects were correlated with soil chemistry. We found that feedback in one explanatory variable, biomass, was significantly related to phylogeny; however this effect was weak and the direction of the feedback effect was opposite to that expected if close phylogenetic relatives were bad neighbors. We also found that soil chemistry was a better predictor of germination and biomass accumulation, but that these relationships showed contrasting patterns.

We found that phylogenetic distance was a weak predictor of plant performance, with plants accumulating more biomass on soils conditioned by families closer to them on the evolutionary tree. This is the reverse of pattern expected for shared pathogens or resource competition among close relatives. Although this might not be surprising as our study only looked at interfamily distances, a recent meta-analysis that included a range of phylogenetic and taxonomic scales showed that even for closely related species, plant–soil feedbacks interactions do not in general correspond to the shared pathogen hypothesis (Mehrabi and Tuck 2015). Interestingly, when soil chemistry was accounted for, no significant effects of phylogeny were detected for feedbacks, whether measured in terms of biomass, growth rate, germination success, or seedling mortality. Taken together, our results do not provide support for the hypothesis that plant performance and survival are improved on soils cultured by species further away from them on the evolutionary tree.

Although conducted on different phylogenetic scales, previous experimental studies investigating the influence of phylogeny on plant–soil feedback did not account for the potential influences of soil chemistry changes in attempt to separate this from shared pathogen-like effects (e.g., Brandt et al. 2009; Burns and Strauss 2011; Liu et al. 2012). The majority of plant–soil feedback experiments also do not determine the net effect of total soil biota and chemistry, either because they exclude some components of the soil community (e.g., McCarthy-Neumann and Kobe 2010a,b), or because soil biota are cultured from inoculations in experiments rather than from field studies using natural biota (Bever 1994; Mills and Bever 1998; but see Burns and Strauss 2011).

We found that soil chemistry was correlated with plant–soil feedback effects. Several manipulative experiments provide evidence in support of a role for chemistry in mediating plant–soil feedback directly (McCarthy-Neumann and Kobe 2010a,b). Other studies suggest that C:N ratios and base cations can affect feedbacks indirectly, by influencing decomposition rates, microbial functioning, and plant productivity (Hobbie 1992; Pérez-Harguindeguy et al. 2000; Ehrenfeld et al. 2005).

The variable effects of soil chemistry observed for seeds, germinants, young seedlings, and old seedlings/mature plants highlight the importance of including multiple life stages in feedback experiments (e.g., Silva Matos et al. 1999; Bell et al. 2006; McCarthy-Neumann and Kobe 2010a,b). For example, the effects of soil chemistry on germination success and accumulation of biomass were inversely related. If similar stage-specific responses occur in the field, then studies targeting brief phases in plant demography may give a misleading indication of the net direction and magnitude of feedback.

In our experiment, we were not able to rule out the effects of species diversity of the parent beds as drivers in the responses, and future work might better understand the influence of the species or functional diversity of parent communities on soil chemistry and feedback responses. A further limitation of the experimental system used here is the use of a restricted number of model species that do not directly mirror natural assemblages. However, our results on using phylogenetic distance as a predictor for plant–soil feedbacks generally support recent meta-analysis on biomass responses that include a larger number of species found in natural assemblages, with higher phylogenetic and life-history resolution (e.g., more species, genera, and families; annuals, perennials, trees, forbs, and shrubs) (Mehrabi and Tuck 2015).

Although the soils in this experiment were high in nutrients (e.g., on average 17% N) and likely to emphasize pathogen over symbiont effects, we did not isolate the soil biota to confirm this. Negative soil feedbacks resulting from changes in soil biota are not just created by pathogens (Bever et al. 1997; van der Putten 2009), and the specificity of interactions of plants with symbionts may not be linearly correlated with feedback responses (Bever 2003). Thus, “black box” experiments such as the one reported here, although demonstrating how patterns of feedback may or may not correspond to mechanisms such as shared pathogens, should be supplemented by further experiments to confirm exactly which organisms are responsible for generating the observed plant responses (Bever et al. 2010; van der Putten et al. 2013). Experimental manipulation that orthogonally treats soil chemistry and soil microbes would be a useful future direction in this respect.

## Supporting Information

Figure S1 Species richness differences between the family beds used in the plant-soil feedback experiments reported in the main text.
