# Supplementary material for: Plant-soil feedbacks from 30-year family-specific soil cultures: phylogeny, soil chemistry and plant life stage
Source: Ecol Evol. 2015 May 22;5(12):2333–9. doi: 10.1002/ece3.1487 (PMC4475366; doi:10.1002/ece3.1487)
Supplement: Figure S1 — Species richness differences between the family beds used in the plant-soil feedback experiments reported in the main text. [file ece30005-2333-sd1.docx]

**Supporting Information**


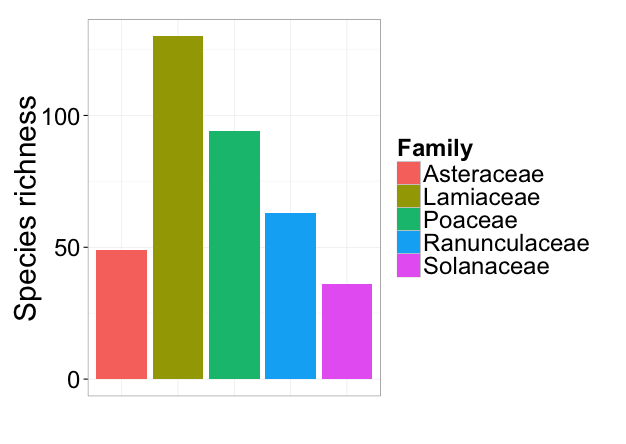


**Figure S1.** Species richness differences between the family beds used in the plant-soil feedback experiments reported in the main text.
